# Genetic Epidemiology of Type 2 Diabetes in Mexican Mestizos

**DOI:** 10.1155/2017/3937893

**Published:** 2017-05-18

**Authors:** Eiralí Guadalupe García-Chapa, Evelia Leal-Ugarte, Valeria Peralta-Leal, Jorge Durán-González, Juan Pablo Meza-Espinoza

**Affiliations:** Facultad de Medicina e Ingeniería en Sistemas Computacionales de Matamoros, Universidad Autónoma de Tamaulipas, Sendero Nacional Km 3, Col. San José, Matamoros, TAMPS, 87349, Mexico

## Abstract

There are currently about 415 million people with diabetes worldwide, a figure likely to increase to 642 million by 2040. In 2015, Mexico was the second Latin American country and sixth in the world in prevalence of this disorder with nearly 11.5 million of patients. Type 2 diabetes (T2D) is the main kind of diabetes and its etiology is complex with environmental and genetic factors involved. Indeed, polymorphisms in several genes have been associated with this disease worldwide. To estimate the genetic epidemiology of T2D in Mexican mestizos a systematic bibliographic search of published articles through PubMed, Scopus, Google Scholar, and Web of Science was conducted. Just case-control studies of candidate genes about T2D in Mexican mestizo inhabitants were included. Nineteen studies that met the inclusion criteria were found. In total, 68 polymorphisms of 41 genes were assessed; 26 of them were associated with T2D risk, which were located in* ABCA1, ADRB3, CAPN10, CDC123/CAMK1D, CDKAL1, CDKN2A/2B, CRP, ELMO1, FTO, HHEX, IGF2BP2, IRS1, JAZF1, KCNQ1, LOC387761, LTA, NXPH1, SIRT1, SLC30A8, TCF7L2, *and* TNF-α* genes. Overall, 21 of the 41 analyzed genes were associated with T2D in Mexican mestizos. Such a genetic heterogeneity compares with findings in other ethnic groups.

## 1. Introduction

Type 2 diabetes (T2D) is a metabolic disorder characterized by impaired glucose uptake in muscle and fat, altered glucose-induced insulin secretion, and increased hepatic glucose production, which lead to hyperglycemia. It is the most common type of diabetes and generally occurs in adults [1]. According to the International Diabetes Federation there are currently around 415 million people with diabetes worldwide, a figure likely to increase to 642 million by 2040 [2]. This disorder accounts for high morbidity and mortality due to complications like renal failure, blindness, amputations, cardiovascular disease, and cerebrovascular events [1]. In 2015 there were approximately 5.0 million deaths by diabetes worldwide [2]. With about 7.3 million patients in 2010 [3], our country was second in Latin America and tenth in the world in prevalence of this disorder [4]. Five years later, the number of diabetic patients was estimated to be 11.5 million and our country ranked sixth in the world [2]. In 2011 most frequent morbidities by T2D were renal failure (24.2%) and peripheral circulatory complications (17.3%), and the mortality rate was 70/100,000 inhabitants (http://fmdiabetes.org/wp-content/uploads/2014/11/diabetes2013INEGI.pdf). The complex etiology of T2D includes factors that influence the risk and evolution of the disease, such as ethnicity, poor alimentation, sedentary lifestyle, obesity, dyslipidemia, and family history [1, 5]. Regarding genetics, worldwide researches have shown association of this disease with numerous allelic variants of nearly 80 candidate genes [6]. The aim of this study is to carry out a literature review about genetic researches conducted in Mexican mestizos for a better understanding of the genetic epidemiology of T2D in our population.

## 2. Methods

A systematic search was done through PubMed, Scopus, Google Scholar, and Web of Science for genetic studies conducted in Mexican mestizo inhabitants with T2D. Key words derived from the phrase “Genetic polymorphisms associated with Diabetes Mellitus type 2 in Mexico, Mexican patients and/or Mexican mestizo” were used. Related terms such as “variants”, “alleles”, and “SNP associated with diabetes, T2DM, or T2D” complemented our search. Just case-control studies of candidate genes performed in Mexican mestizos resident in the country were included. Researches conducted in Mexican native populations were excluded, as well as those done in patients with metabolic syndrome. In surveys that included both patients with metabolic syndrome and patients with T2D, only cases with T2D were registered. Although in the selected studies different models of genotype analyses were used (recessive, dominant, or codominant), solely comparisons between allele frequencies were considered in our review. In studies without described odds ratio (OR), unadjusted OR were estimated from the reported allele or genotype frequencies. Allele comparisons were performed by 2 × 2 contingency tables [Yates' correction chi-square test (http://vassarstats.net/odds2x2.html)] and genotypes were contrasted by chi-square test (https://ihg.gsf.de/cgi-bin/hw/hwa2.pl). In both comparisons, OR were estimated using 95% confidence intervals (95% CI). A *p* ≤ 0.05 defined a significant association.

Whenever a polymorphism was analyzed in different studies, data were combined and unadjusted OR for alleles were calculated using a 2 × 2 contingency table (Yates' correction chi-square test). However, studies with suspicion of overlapping of patients were not included in this analysis.

## 3. Results

In total, 19 case-control studies on the possible association of genetic polymorphisms with T2D in Mexican mestizos resident in the country were included [7–25]. Altogether, 68 polymorphisms of 41 genes were assessed (Table 1). Of them, 25 were associated with an increased risk for T2D and they were located in 20 genes, namely,* ABCA1, ADRB3, CAPN10, CDC123/CAMK1D, CDKN2A/2B, CRP, ELMO1, FTO, HHEX, IGF2BP2, IRS1, JAZF1, KCNQ1, LOC387761, LTA, NXPH1, SIRT1, SLC30A8, TCF7L2, *and* TNF-α*. Among the variants that showed association there were 4/20 amino acid substitutions, 13/30 intronic sites, 6/10 of promoter region or 5′-flanking region or upstream of gene, 1/2 intergenic regions, and 2/6 of 3′-untranslated or 3′-flanking region of gene. On the other hand, 12 polymorphisms were analyzed by different authors, and concordance was observed in most of them, except for rs3842570* (CAPN10)* [11, 13, 14], rs13266634* (SLC30A4)* [8, 17], rs7903146* (TCF7L2)* [8, 10, 12, 22], and rs1800629* (TNF-α)* [20, 24, 25]. Eleven of these polymorphisms were pooled and analyzed as shown in Table 2. Note that rs4994* (ADRB3)* was discarded of this analysis (suspicion of overlap of [9, 10]). Similarly, data by Cruz et al. [10] for rs7903146 and rs12255372 of* TCF7L2* were not considered (possible overlapping with the study by Martínez-Gómez et al. [12]). Thus, the 3R allele of rs3842570, which was associated with T2D in a small sample, did not seemingly confer susceptibility to the disease; in contrast, the C allele of rs7754840* (CDKAL1)*, which evidenced no risk in independent studies, showed association with T2D. Including this allele, a total of 26 polymorphisms and 21 genes were associated with T2D in Mexican mestizos.

## 4. Discussion

This review about genetics of T2D in Mexican mestizo subjects shows that 26 polymorphisms distributed in 21 genes are associated with this disease, so T2D has a high heterogeneity in our population, the same as that in other ethnic groups. Therefore, in some individuals alleles of certain genes are involved, while in others subjects are implicated variants of different genes. A previous conclusion that T2D in Mexican mestizos is genetically homogeneous was based on an analysis of three genetic markers [26] and here appears untenable. Though the Mexican mestizo population has a European genetic ancestry near 30% [27], not all the alleles conferring diabetes risk in Europeans are associated with T2D in our population [8]. These variations could be related to genetic background, differences in clinical classifications, sample size, selection and analysis criteria, and environmental factors such as obesity, lifestyle, and diet. On the other hand, researches in several ethnic groups have shown association of T2D with genes not yet analyzed in Mexican population [5, 6, 28–32]. It would be important to carry out the analysis of such genes to determine whether these variants are also associated with T2D in Mexican patients and increase the knowledge about the genetic epidemiology of this disorder in our country.

Regarding Mexican studies, an increased risk was detected when analysis was performed adjusting covariates. For instance, Cruz et al. observed an additive effect in the T2D risk when they considered variables such as age, education, sex, body mass index, and ancestry [10]. Gamboa-Meléndez et al. reported association with T2D for the polymorphisms rs7923837* (HHEX)*, rs4402960* (IGF2BP2)*, and rs2237892* (KCNQ1)* only when ancestry was adjusted [8]. For the polymorphisms rs864745* (JAZF1)* and rs757705* (NXPH1)*, the analysis stratified by ancestry did not show significant differences, whereas an association was observed in the comparison without such an adjustment. In addition, they found association for rs7903146* (TCF7L2)* and rs7754840* (CDKAL1)* just in early-onset T2D [OR = 1.39 (1.04–1.85), *p* = 0.024] and in nonobese T2D patients [OR = 1.25 (1.06–1.49), *p* = 0.009], respectively. Another study found a lower OR when the analysis was adjusted by sex, body mass index, and family history of T2D for three polymorphisms of* IRS1* in a dominant model [19].

The reported association of rs3842570* (CAPN10)* [14], rs909253* (LTA)* [20], and rs1800629* (TNF-α)* [24] with T2D should be interpreted with caution given the small sample sizes and poor statistical power. With respect to the rs1345365 polymorphism* (ELMO1)*, the authors reported a protector effect for the A allele [OR = 0.65 (0.55–0.78),* p* < 0.001] [16]. But in our analysis we took as reference the A allele, as it is the most common; thus, the G allele showed association with T2D [OR = 1.37 (1.02 to 1.84), *p* = 0.035].

Since T2D is a complex disorder and several genes are implicated in its etiology and evolution, the identification of risk alleles could be useful, because if the involved genes and their function are known, it is more probable to achieve prevention, treatment, prognosis, and/or cure of the disease. Complications could also be prevented or treated better [29, 33]. However, published studies demonstrate that genetic screening for the prediction of T2D in high risk subjects is currently of little value in clinical practice. Actually, genetic risks are difficult to calculate because several alleles could contribute to an additive effect to T2D susceptibility [34], not to mention the diverse environmental factors involved. Although some of these genes are implicated in the glucose and fat metabolism, *β*-cell function, and sensitivity and secretion of insulin [29, 35], how some of their variants increase the T2D risk remains to be elucidated [29]. Anyway, it is fundamental to analyze the genetic epidemiology of this disease in each population because of the underlying differences in genetic background and lifestyle among ethnic groups. So, it is possible that polymorphisms associated with T2D in some races do not show association in others. Genome-wide association studies will ultimately precise the genetic landscape.

## Figures and Tables

**Table 1:** Analyzed genes in studies about type 2 diabetes conducted in Mexican mestizos.

Gene	Chrom	dbSNP loc	Change	Effect	*n* ^a^; *n*^b^	OR (95% CI)	*p*	Reference
*ABCA1*	9q31	rs9282541	C/**T**	R/C	244; 202	**2.50 (1.48–4.24)**	**0.001**	[7]
		rs2000069	C/**T**	Intronic	244; 202	1.08 (0.82–1.42)^c^	0.58	[7]
		rs2230806	G/**A**	R/K	244; 202	1.17 (0.89–1.55)^c^	0.27	[7]
		rs2487037	C/**T**	Intronic	244; 202	1.06 (0.79–1.43)^c^	0.71	[7]
		rs3818689	G/**C**	Intronic	244; 202	0.94 (0.52–1.68)^c^	0.82	[7]

*ADAMTS9*	3p14	rs4607103	C/**T**	Intronic	1027; 990	1.05 (0.91–1.20)^d^	0.521	[8]

*ADRB1*	10q25	rs1801253	C/**G**	R/G	501; 552	0.79 (0.61–1.02)^c^	0.07	[9]

*ADRB3*	8p11	rs4994	C/**T**	W/R	519; 547	**1.69 (1.37–2.09)** ^c^	**0.0001**	[10]
		rs4994	C/**T**	W/R	501; 552	**1.34 (1.10–1.64)** ^c^	**0.004**	[9]

*ARHGEF11*	1q21	rs945508	G/**A**	R/H	868; 504	0.91 (0.76–1.09)^e^	0.319	[8]

*CAPN10*	2q37	rs3792267	G/**A**	Intronic	132; 112	0.97 (0.66–1.42)^c^	0.86	[11]
		rs3792267	G/**A**	Intronic	719; 746	1.11 (0.95–1.29)^c,f^	0.20	[12]
		rs3792267	G/**A**	Intronic	211; 152	0.91 (0.66–1.26)	0.56	[13]
		rs3842570	2R/**3R**	Intronic	132; 112	0.97 (0.68–1.40)^c^	0.89	[11]
		rs3842570	2R/**3R**	Intronic	43; 64	**1.81 (1.03–3.18)** ^**c**^	**0.038**	[14]
		rs3842570	2R/**3R**	Intronic	211; 152	0.75 (0.55–1.02)	0.06	[13]
		rs5030952	C/**T**	Intronic	132; 113	0.85 (0.56–1.29)^c^	0.45	[11]
		rs5030952	C/**T**	Intronic	211; 152	1.35 (0.89–2.06)	0.16	[13]
		rs2975760	T/**C**	Intronic	134; 113	**2.72 (1.16–6.35)**	**0.017**	[11]

*CAPN10*	2q37	rs7607759	A/**G**	T/A	127; 110	2.27 (0.98–5.25)^c^	0.051	[11]

*CDC123/CAMK1D*	10p13	rs12779790	A/**G**	Intergenic	1027; 990	**1.24 (1.05–1.47)** ^d^	**0.013**	[8]

*CDKAL1*	6p22	rs10946398	A/**C**	Intronic	519; 547	1.09 (0.91–1.32)^c^	0.337	[10]
		rs9465871	**C**/T	Intronic	519; 547	1.04 (0.85–1.26)^c^	0.718	[10]
		rs7754840	**C**/G	Intronic	519; 547	1.08 (0.89–1.29)^c^	0.438	[10]
		rs7754840	**C**/G	Intronic	1027; 990	1.13 (0.98–1.30)^d,g^	0.081	[8]

*CDKN2A/2B*	9p21	rs10811661	C/**T**	Upstream	1027; 990	**1.42 (1.15–1.75)** ^d^	**0.001**	[8]

*CRP*	1q23	rs1130864	C/**T**	3′-UTR	166; 130	**1.59 (1.15–2.22)** ^c,h,i^	**0.005**	[15]
		rs1205	G/**A**	3′-UTR	166; 130	0.82 (0.59–1.14)^c,h,i^	0.24	[15]
		rs2794521	A/**G**	5′-flanking	166; 130	**1.97 (1.15–3.38)** ^c,h,i^	**0.012**	[15]
		rs3093062	G/**A**	Promoter	166; 130	**3.49 (0.98–12.4)** ^c,h,i^	**0.039**	[15]

*ELMO1*	7p14	rs1345365	A/**G**	Intronic	148; 269	**1.37 (1.02–1.84)** ^c,h,i^	**0.035**	[16]

*ENPP1*	6q23	rs1044498	A/**C**	K/Q	519; 547	0.94 (0.76–1.16)^c^	0.577	[10]

*EXT2*	11p11	rs3740878	**A**/G	Intronic	455; 234	0.83 (0.65–1.05)	0.054	[17]

*FTO*	16q12	rs8050136	**A**/C	Intronic	868; 504	0.90 (0.74–1.09)^e^	0.278	[8]
		rs9939609	**A**/T	Intronic	519; 547	**1.25 (1.02–1.54)** ^c^	**0.027**	[10]

*HHEX*	10q23	rs5015480	**C**/T	Upstream	519; 547	0.96 (0.80–1.14)^c^	0.631	[10]
		rs1111875	**C**/T	3′-flanking	1027; 990	1.01 (0.89–1.16)^d^	0.859	[8]
		rs1111875	**C**/T	3′-flanking	455; 234	1.12 (0.88–1.44)	0.27	[17]

*HHEX*	10q23	rs7923837	A/**G**	3′-flanking	868; 504	**1.21 (1.02–1.44)** ^k^	**0.025**	[8]

*HMOX1*	22q12	rs2071749	A/G	Promoter	614; 956	0.98 (0.84–1.14)^c^	0.76	[18]

*IGF2BP2*	3q27	rs4402960	G/**T**	Intronic	868; 504	**1.24 (1.01–1.53)** ^j^	**0.042**	[8]

*IRS1*	2q36	rs1801278	G/**A**	G/R	719; 746	**2.04 (1.41–2.96)** ^c,f^	**<0.001**	[12]
		rs1801278	G/**A**	G/R	444; 444	**3.22 (1.99–5.20)**	**0.001**	[19]
		rs1801276	C/**G**	P/A	444; 444	0.98 (0.72–1.32)	0.83	[19]
		rs3731594	G/**A**	N/D	444; 444	0.83 (0.42–1.66)	0.47	[19]
		rs1801108	**G**/C	R/P	444; 444	1.07 (0.85–1.34)	0.40	[19]

*JAZF1*	7p15	rs864745	**T**/C	Intronic	868; 504	**1.24 (1.04–1.47)** ^k^	**0.015**	[8]

*KCNJ11*	11p15	rs5215	C/**T**	V/I	519; 547	1.03 (0.87–1.23)^c^	0.729	[10]
		rs5210	**A**/G	3′-UTR	519; 547	1.03 (0.86–1.23)^c^	0.764	[10]
		rs5219	C/**T**	E/K	1027; 990	1.10 (0.96–1.26)^d^	0.154	[8]

*KCNQ1*	11p15	rs2237892	**C**/T	Intronic	868; 504	**1.36 (1.13–1.64)** ^k^	**0.001**	[8]

*LEPR*	1p31	rs1137100	**A**/G	K/R	519; 547	1.00 (0.84–1.21)^c^	0.92	[10]

*LOC387761*	11p12	rs7480010	A/**G**	Intronic	455; 234	**1.43 (1.05–1.94)**	**0.006**	[17]

*LTA*	6p21	rs909253	**A**/G	Intronic	51; 48	**1.98 (1.02–3.8)** ^c^	**0.041**	[20]

*MGEA5*	10q24	MGEA5-14	A/**T**	Intronic	271; 244	1.60 (0.52–4.86)	0.404	[21]

*NOTCH2*	1p11	rs10923931	G/**T**	Intronic	1027; 990	1.04 (0.82–1.32)^d^	0.731	[8]

*NQO1*	16q22	rs1800566	C/**T**	P/S	623; 993	0.98 (0.85–1.13)^c^	0.76	[18]

*NRF2*	2q31	rs2364723	C/**G**	Intronic	625; 992	0.91 (0.79–1.05)^c^	0.18	[18]

*NRF2*	2q31	rs6721961	C/**A**	Promoter	623; 989	0.89 (0.74–1.06)^c^	0.18	[8]

*NXPH1*	7p22	rs757705	A/**G**	Intronic	868; 504	**1.25 (1.05–1.48)** ^k^	**0.01**	[8]

*PPARG*	3p25	rs1801282	**C**/G	P/A	719; 746	1.00 (0.81–1.24)^c,f^	1.00	[12]
		rs1801282	**C**/G	P/A	1027; 990	1.10 (0.90–1.34)^d^	0.342	[8]
		rs17793693	**A**/C	Intronic	519; 547	1.09 (0.91–1.31)^c^	0.329	[10]

*RALGPS2*	1q25	rs2773080	**A**/G	Intronic	868; 504	0.90 (0.74–1.10)^e^	0.315	[8]

*RORA*	15q22	rs7164773	C/**T**	Intronic	868; 504	1.08 (0.91–1.28)^e^	0.357	[8]

*SIRT1*	10q21	rs3758391	C/**T**	Upstream	519; 547	**1.29 (1.08–1.54)** ^c^	**0.004**	[10]

*SLC30A4*	8q24	rs13266634	**C**/T	R/W	455; 234	1.01 (0.76–1.33)	0.92	[17]
		rs13266634	**C**/T	R/W	1027; 990	**1.22 (1.05–1.41)** ^d^	**0.009**	[8]

*TCF7L2*	10q25	rs7903146	C/**T**	Intronic	868; 504	1.04 (0.84–1.28)^e,l^	0.735	[8]
		rs7903146	C/**T**	Intronic	200; 200	**1.84 (1.05–3.20)** ^c,m^	**0.04**	[12]
		rs7903146	C/**T**	Intronic	519; 547	**1.48 (1.18–1.86)** ^c^	**0.0007**	[10]
		rs7903146	C/**T**	Intronic	283; 271	1.25 (0.92–1.70)	0.16	[22]
		rs12255372	G/**T**	Intronic	200; 200	**1.83 (1.21–2.76)** ^c,m^	**0.006**	[12]
		rs12255372	G/**T**	Intronic	281; 268	**1.78 (1.11–2.88)**	**0.017**	[22]
		rs12255372	G/**T**	Intronic	519; 547	**1.37 (1.06–1.76)** ^c^	**0.014**	[10]
		DG10S478	STR CACA	Intronic	282; 274	**1.62 (1.02–2.57)**	**0.041**	[22]

*TLR2*	4q32	rs5743708	G/**A **	R/Q	321; 538	0.41 (0.04–3.7)	0.40	[23]

*TLR4*	9q33	rs4986790	A/**G**	D/G	321; 538	1.39 (0.42–4.56)	0.58	[23]
		rs4986791	C/**T**	T/I	321; 538	1.01 (0.32–3.18)	0.98	[23]

*TNF-α*	6p21	rs1800629	-308G/**A**	Upstream	51; 48	0.76 (0.31–1.85)^c,n^	0.55	[20]
		rs1800629	-308G/**A**	Upstream	95; 87	**4.66 (1.73–12.5)** ^c^	**0.001**	[24]
		rs1800629	-308G/**A**	Upstream	259; 645	1.25 (0.83–1.87)	0.29	[25]
		rs361525	-238G/**A**	Upstream	259; 645	**1.57 **(**1.07–2.29**)	**0.018**	[25]

*TSPAN8/LGR5*	12q14–q21	rs7961581	C/**T**	Intergenic	868; 504	0.93 (0.73–1.17)^e^	0.516	[8]

*TXNIP*	1q21	rs7211	C/**T**	3′ UTR	623; 969	0.97 (0.82–1.14)	0.67	[18]

*UBQLNL*	11p15	rs979752	**C**/T	Upstream	868; 504	1.04 (0.84–1.30)^e^	0.70	[8]

Chrom: chromosome. Risk alleles are marked in bold. *n*^a^; *n*^b^. Sample for cases and controls, respectively. ^c^Conventional OR (unadjusted) was assessed by us from allele or genotype frequencies reported. ^d^Largest *n* was registered. ^e^Test without ancestry correction was considered. ^f^Combined datasets were registered. ^g^Risk was only observed in nonobese T2D patients (OR = 1.25; *p* = 0.009). ^h^Only Genotypes of T2D patients and healthy controls were used in our analysis. ^i^Assessment derived from the sum of T2D patients (obese and nonobese). ^j^The authors reported a protector effect for the A allele (OR = 0.65; *p* < 0.001), but in our estimation we took as reference the A allele, since it is the most common. ^k^Significant analysis with ancestry correction was taken. ^l^Association was only found in early-onset T2D (OR = 1.39; *p* = 0.024). ^m^Just the population of Guerrero was recorded due to possible overlapping of the patients from the Mexico City with [10]. ^n^The G allele was assessed as risk by the authors; but in our analysis we took the A allele, the same as that in previous studies.

**Table 2:** Analysis of SNPs studied by two or more groups in Mexican mestizos.

Gene	dbSNP loc	Cases (allele)	Controls (allele)	Risk allele frequency (%)		OR (95% CI)	*p* ^*∗*^ value	Reference
				Cases	Controls			
*CAPN10*	rs3792267	1086	928	29.5	28.7	1.06 (0.87–1.29)	0.56	[11–13]
*CAPN10*	rs3842570	772	656	61.7	62.5	0.96 (0.78–1.20)	0.74	[11, 13, 14]
*CAPN10*	rs5030952	686	530	19.0	18.3	1.04 (0.78–1.40)	0.78	[11, 13]
*CDKAL1*	rs7754840	3092	3074	31.4	29.1	**1.12 (1.00–1.25)**	**0.044**	[8, 10]
*HHEX*	rs1111875	2974	2448	62.8	61.7	1.05 (0.94–1.17)	0.43	[8, 17]
*IRS1*	rs1801278	2326	2380	6.6	2.8	**2.45 (1.83–3.28)**	**<0.0001**	[12, 19]
*PPARG*	rs1801282	3492	3472	87.3	86.7	1.05 (0.91–1.21)	0.51	[8, 12]
*SLC30A4*	rs13266634	2964	2448	76.3	73.0	**1.19 (1.05–1.35)**	**0.005**	[8, 17]
*TCF7L2*	rs7903146	3740	3042	17.8	14.1	**1.32 (1.16–1.50)**	**<0.0001**	[8, 12, 22]
*TCF7L2*	rs12255372	2476	2586	18.3	11.6	**1.40 (1.19–1.65)**	**<0.0001**	[12, 22]
*TNF-α*	rs1800629	810	1560	11.5	6.2	**1.96 (1.45–2.64)**	**<0.0001**	[20, 24, 25]

^*∗*^Yates' correction chi-square test.
